# Coupling of Event-Related Potential and Pupil Dilation as a Compensatory Marker of Executive Attention in Traumatic Brain Injury

**DOI:** 10.1177/2689288X251370997

**Published:** 2025-08-26

**Authors:** Giacomo Scanavini, Isabelle Martin, Ludvik Alkhoury, Ana Radanovic, Yakira Tepler, Abhishek Jaywant, N. Jeremy Hill, Tracy Butler, Keith W. Jamison, Amy Kuceyeski, Nicholas D. Schiff, Sudhin A. Shah

**Affiliations:** ^1^ Department of Radiology, Weill Cornell Medicine; New York, New York, USA.; ^2^ Department of Psychiatry, Weill Cornell Medicine; New York, New York, USA.; ^3^ Department of Rehabilitation Medicine, Weill Cornell Medicine; New York, New York, USA.; ^4^ National Center for Adaptive Neurotechnologies, Stratton VA Medical Center; Albany, New York, USA.; ^5^ Department of Electrical and Computer Engineering, University at Albany - State University of New York; Albany, New York, USA.; ^6^ Department of Computational Biology, Cornell University; Ithaca, New York, USA.; ^7^ Department of BMRI & Neurology, Weill Cornell Medicine; New York, New York, USA.

**Keywords:** adult brain injury, electrophysiology, pupillometry, traumatic brain injury

## Abstract

Traumatic brain injury (TBI) impairs attention and executive function, often through disrupted coordination between cognitive and autonomic systems. While electroencephalography (EEG) and pupillometry are widely used to assess neural and autonomic responses independently, little is known about how these systems interact in TBI. Understanding their coordination is essential to identify compensatory mechanisms that may support attention under conditions of neural inefficiency. In this study, we examined pupil dilation during the Attention Network Test in individuals with TBI (*n* = 25) and controls without brain injury (*n* = 45). TBI participants exhibited preserved accuracy but slower reaction times (RTs), suggesting increased cognitive effort. Paradoxically, this effort was not reflected in heightened pupil dilation. Instead, pupil responses were attenuated, suggesting impaired recruitment of the locus coeruleus–norepinephrine system and possible autonomic dysregulation. We further assessed the relationship between simultaneously recorded pupillary responses and visual evoked responses in a subset of those in whom both measures were available (*n* = 23, TBI; *n* = 35, controls). Crucially, while both pupil dilation and amplitude of the visual P3 event-related potential were reduced in TBI, these measures showed a positive correlation across participants with TBI; this was absent in controls. Our results suggest that TBI may induce a compensatory coupling between cortical and autonomic systems to sustain cognitive performance despite underlying dysfunction. Positive correlation between pupil dilation and event-related potential suggest a role for arousal dysregulation in subjects with TBI. Our findings provide new evidence for altered EEG-pupil dynamics in TBI and highlight the potential of combining cortical and autonomic measures as a multimodal biomarker for tracking recovery, stratifying injury severity, and guiding individualized rehabilitation strategies.

## Introduction

Traumatic brain injury (TBI) impairs executive function, leading to difficulties with sustained attention, cognitive flexibility, and decision-making.^1^ While these deficits are well-documented, their neurophysiological underpinnings are not fully understood. Autonomic dysfunction is a potential factor, resulting from damage to brain regions that regulate involuntary physiological processes such as heart rate, blood pressure, and arousal.^2^ These disruptions may impair cognitive performance by affecting stress regulation and cognitive load management, exacerbating attention and executive function deficits.^3^ Investigating how cognitive and autonomic systems interact in TBI could provide crucial insights into the compensatory mechanisms the brain employs to sustain function following injury.^4^ Such findings could inform better diagnostic markers, prognostic assessments, and rehabilitation strategies to optimize cognitive recovery.

Pupil dilation, a well-established marker of sympathetic activity and cognitive effort,^5,6^ is driven by the locus coeruleus–norepinephrine (LC-NE) system, which modulates attention and arousal.^7^ In healthy individuals, pupil responses reflect real-time mental effort, resource allocation, and cognitive flexibility.^5,8^ In individuals with TBI, pupillary responses are less well-characterized, but some studies suggest that reduced dilation may reflect autonomic dysregulation and reduced LC-NE engagement.^9,10^

Event-related potentials (ERPs), recorded in response to stimulus processing, have been widely studied in TBI, with findings indicating attenuated visual P3 responses, which may reflect decreased neural efficiency and impaired attentional resource allocation.^11^ Recent work has demonstrated that electrophysiological markers of executive attention, particularly in midline frontal regions, are attenuated in TBI, mirroring response patterns observed in older adults.^12^ Specifically, individuals with moderate-to-severe TBI exhibit reduced frontal cortical activation and diminished differentiation between low- and high-demand trials, suggesting that TBI disrupts key cortical circuits supporting executive attention.

While both pupil dilation and P3 independently relate to task engagement, studies using the oddball paradigm have shown they do not consistently relate to each other within or across subjects.^13–15^ In contrast, a study using a high-demand emotional conflict task has reported a relationship within and across subjects.^16^

The current study examines the relationship between electroencephalography (EEG) (specifically, visual P3 amplitude and latency) and pupil dilation during the Attention Network Test (ANT)^17^ in individuals with TBI and controls. Despite the well-documented disruptions in both EEG and autonomic function in TBI, to the best of our knowledge, no prior studies have explored the association between EEG and pupillometry in the context of executive attention in TBI. Prior research using the ANT suggests that TBI participants maintain accuracy but exhibit prolonged RTs, indicating increased cognitive effort and slowed processing.^12,18–20^ Given that TBI disrupts both cortical and autonomic regulation, understanding how these systems interact could reveal compensatory mechanisms or biomarkers of cognitive dysfunction that could improve clinical assessments and guide targeted rehabilitation strategies.

## Methods

### Participants

Participants were drawn from a larger longitudinal study using the ANT, neuropsychological clinical measures, EEG, eye-tracking, Positron Emission Tomography, and Magnetic Resonance Imaging (MRI); some parts of this dataset have previously been published.^12,20,21^ In the current study, we included individuals with a history of TBI along with controls (see Table 1). Participants with TBI were 7 ± 3 (S.D.) months post-injury. They had either complicated mild injury with intracranial neuroimaging abnormalities on acute Computed Tomography/MRI scan, or moderate-to-severe brain injury with at least one of the following: post-traumatic amnesia for more than 24 h, loss of consciousness for at least 30 min, and Glasgow Coma Scale (GCS)^22^ in the emergency department lower than 13. All participants spoke English, were able to provide informed consent, and complete questionnaires and cognitive testing. In addition, all had normal or corrected-to-normal vision. We excluded participants who had a history of alcohol or substance use disorder, visual, auditory, pre-existing neurological disorders, and/or motor impairments that would interfere with cognitive testing. All controls had no prior brain injury or neurological disorders. All procedures were approved by the Institutional Review Board of Weill Cornell Medicine.

**Table 1.** Demographic Information for the Participants With and Without Traumatic Brain Injury Included in the Stand-Alone Pupil Data Analysis and Combined Pupil and EEG Data Analysis

	Pupil data		Pupil + EEG data	
	Control	TBI	Control	TBI
Participants				
Sample size [*n*]	45	25	35	23
Female [*n* (%)]	16 (36%)	9 (36%)	13 (37%)	9 (39%)
Age at session [years ± S.D.]	40.5 ± 15.2	48.1 ± 19.1	41.2 ± 14.5	48.0 ± 18.8
Injury severity (GCS)				
Complicated mild, GCS >12 [*n* (%)]	N/A	16 (64%)	N/A	14 (61%)
Moderate, 9 ≤ GCS ≤12 [*n* (%)]	N/A	3 (12%)	N/A	3 (13%)
Severe, GCS <9 [*n* (%)]	N/A	6 (24%)	N/A	6 (26%)

TBI, traumatic brain injury; EEG, electroencephalography; GCS, Glasgow Coma Scale.

### Assessment

The ANT is a widely used paradigm for assessing the efficiency of three attentional networks: alerting, orienting, and executive control.^17^ In each trial, participants fixate on a central cross before identifying the direction of a target arrow, which is flanked by distractor arrows in either a congruent or incongruent arrangement. Trials follow a structured sequence: a fixation phase of random duration (400–1600 ms), a cue phase (100 ms) where either no-cue, a non-spatial center-cue, or a spatial-cue is presented, another brief fixation (400 ms), followed by the target phase (maximum allowable response time of 1700 ms). The trial concludes with an inter-trial fixation period, ensuring a fixed total trial duration of 4000 ms. Participants first complete a training block (24 trials) with feedback after each trial (reaction time and accuracy), followed by three experimental blocks (96 trials each) without feedback. RTs from correct trials, only, are used to compute attentional network effects as follows:

- Alerting = RT(no-cue) − RT(center-cue);
- Orienting = RT(center-cue) − RT(spatial-cue);
- Executive Control = RT(incongruent) − RT(congruent).

Higher alerting and orienting scores indicate greater cue utilization, while higher executive control scores reflect greater difficulty resolving conflict.

### Apparatus

The ANT stimuli were presented using Eprime 2.0^23^ on a 22-inch Liquid-crystal display monitor (resolution of 1920 × 1080 pixels). Stimuli were displayed in black, on a white background. A 2-button mouse was held by both hands throughout the session, and the participants were instructed to use their left and right thumbs to indicate the direction of the target arrow. Eye-tracking was conducted with the EyeLink 1000 Plus (SR Research) in remote mode at a sampling rate of 500 Hz. The dominant eye was tracked with a “target” sticker placed over the eyebrow. A 3- or 9-point calibration was conducted at the beginning of each session.

Simultaneous EEG data were recorded at 1000 Hz sampling rate with a 128-channel HydroCel Geodesic Sensor Net (Magstim-EGI, Eugene, OR, USA).^24^ The impedance was measured to be below 50 kΩ at the beginning of the recording session. The experiment was conducted in a quiet room with white neon lighting; participants were seated at approximately 70 cm from the eye-tracking device (placed in front of the monitor) and 75 cm from the monitor.

### Availability of pupil data

Eye-tracking data were collected in *n* = 58 controls and *n* = 38 participants with TBI (*n* = 17 and *n* = 22 had two sessions, respectively). Given the lack of a chin-resting system, data quality was assessed for each available dataset (up to two for each participant) with a goal of identifying a single session per individual with high-quality pupil data. If both sessions met the criteria for pupil quality (see Pupil data processing and quality checks below), we used the first session. We rejected *n* = 13 control and *n* = 13 TBI participants. The final numbers included in the pupil analysis were *n* = 45 controls and *n* = 25 TBI.

### Availability of EEG data

For participants with adequate pupil data, we determined the quality of simultaneously recorded EEG data. We rejected *n* = 10 control and *n* = 2 TBI participants from EEG data analysis due to lack of usable data (see EEG data processing and quality checks). The final number of participants for whom both the pupil and EEG data were acceptable was *n* = 35 controls and *n* = 23 TBI.

### Data processing

#### Extraction of pupil data

The standard output file (.edf, EyeLink Data Format) was converted to American Standard Code for Information Interchange (.asc) using the software EDF2ASC provided by SR Research. The .asc file was parsed using a custom Python script to extract eye-tracking information (e.g., gaze coordinates and pupil area) and events (e.g., blinks and saccades).

#### Pupil data processing and quality checks

The following steps were conducted for each dataset available (up to two datasets per participant). Our goal was to identify a single session per participant.

- Visual inspection of each trial’s X and Y gaze coordinates, between target onset and response, was used to identify calibration issues; *n* = 1 control was excluded.
- To ensure the quality of trial-by-trial data (4 s per trial), we rejected the following: 1. Trials with incorrect or missing behavioral response. 2. Trials with missing eye-tracking data. 3. Trials that failed blink interpolation. As blinks result in missing data, we first computed the derivative of pupil size to detect the location of abrupt changes. To account for edge effects, we padded with 160 ms of zeros before and after the identified locations, effectively extending the blink window (see differences between the original signals in Supplementary Fig. S1.A and the processed signals in Supplementary Fig. S1.B). A linear interpolation was implemented to fill in the missing samples. If the blink events occurred at the start or end of the trial, a flat constant value was used to match the first or last available data point (see Supplementary Fig. S1.C). Trials with 45% or more interpolated samples were rejected. In addition, we visually inspected all trials to identify failed interpolations. A total of *n* = 6 controls and *n* = 6 TBI were excluded because their data had fewer than 15% usable trials per cue and target condition.
- Pupil dilation was computed on a trial-by-trial basis. Each trial was aligned to 500 ms before target appearance; this time point (set as 0 s) coincides with the onset of spatial or center-cues, and is referred to as *cue-aligned* in the manuscript. For baseline correction, we used the 400 ms preceding cue onset. The average pupil dilation during this baseline window was subtracted from the entire trial, setting each trial to start at approximately 0 AU (arbitrary units) prior to cue onset (see baseline corrected signals in Supplementary Fig. S1.D). Next, trials were grouped by the three cue types and two target types. We visually inspected the average pupil response for each participant to identify and exclude cases with a inclined baseline trend, as these could distort subsequent pupil dilation measures (*e.g.*, peak amplitude). As a result, we excluded an additional *n* = 6 controls and *n* = 6 TBI.
- We performed a separate alignment of trials to the time of response, referred to as *response-aligned* in the text.
- For each alignment, trials with the same condition were aggregated via arithmetic mean, and pupil dilation amplitude and latency were extracted by considering the location of the maximum amplitude in the time window post-target onset.
- Similarly, group-level pupil dilations were extracted via arithmetic mean.

Overall, the pupil data analysis includes a single session from *n* = 45 control and *n* = 25 TBI participants.

#### EEG data processing and quality checks

The following steps were conducted for each dataset available:

- EEG data were rereferenced to the average of the mastoids and bandpass filtered between 1 and 10 Hz. A visual inspection was performed to manually identify and flag bad time segments (*e.g.*, due to the presence of muscle artifacts) and channels. This step was done without overlaying experimental annotations to minimize selection bias.
- Data were segmented into 1.9 s epochs, 0.9 s before and 1 s after target onset. The 0.9 s pre-target window included 400 ms before cue onset, plus 500 ms between cue and target onset. The former segment was used for baseline correction because: 1. As with the pupil data analysis, it is the longest time frame consistently present across all trials, and 2. It represents ∼20% of the epoch’s duration.
- Each epoch was labeled by its trial type and grouped according to the cue types (no, center, spatial) and target types (congruent, incongruent). Epochs corresponding to trials with incorrect or missing responses, or those previously rejected due to poor pupil data, were similarly excluded to match the pupil dataset. The remaining epochs were downsampled to 120 Hz for faster processing.
- The Random Sample Consensus algorithm^25^ was applied to detect and interpolate bad channels. Then, Autoreject^26^ was used to identify and remove, within each group, epochs affected by artifacts.
- For each participant, we computed ERPs by aggregating the epochs with the same conditions. A weighted average was calculated at each time sample, with weights derived from a probability density function built via kernel density estimate.^27,28^
- The uncertainty at each time sample was estimated via the bootstrap method by resampling with replacement 1000 times.
- P3 ERP amplitude and its uncertainty were extracted by identifying the maximum amplitude at electrode Pz within the 300–700 ms post-target window. The amplitude was then computed as the average over a 125 ms window defined as (−42 ms, +83 ms) from the maximum point. The uncertainty was obtained by propagating the uncertainties of the time samples used for the average.
- P3 ERP latency was extracted by considering the location of the maximum amplitude. The uncertainty was calculated as half the interval between the left and right points at which the ERP signal intersected the maximum amplitude minus its uncertainty.
- To compute group-level evoked potentials, participant-level ERPs were aggregated using the previously mentioned weighted average. Its uncertainty was also estimated via the bootstrap method.
- Individual-level ERPs were transformed using signal-to-noise ratio for the purpose of evaluating linear relationships with other parameters (e.g., pupil dilation).

Due to non recorded or poor quality EEG data, assessed by visual inspection, the total number of participants was reduced by *n* = 10 controls and *n* = 2 TBI, leaving *n* = 35 and *n* = 23, respectively.

### Statistical analysis

For statistical evaluations, two-sample two-tailed paired *t*-tests^29^ are used when comparing the means of variables from different trial types within the same cohort (e.g., pupil dilation amplitude for congruent and incongruent trials). Two-sample Welch’s *t*-tests^30^ are used when comparing the means of variables across different cohorts; this is to account for differences in sample sizes and to be conservative regarding the variances of the two groups (e.g., controls vs. TBI RTs for congruent target trials).

**FIG. 1.**
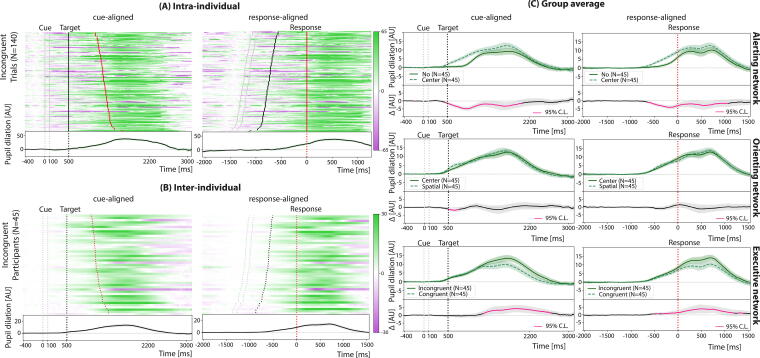
Pupil dilation across cue- and response-aligned trials in control participants. **(A)** Raster plots with pupil dilation traces from a single control. Incongruent target trials, sorted according to increasing reaction time from top to bottom, are shown. **(B)** Raster plots with mean pupil dilation traces for all control participants included in the analysis, *n* = 45. Participants are sorted according to increasing reaction time in incongruent trials from top to bottom. **(C)** Group average pupil dilation comparisons for the control cohort. Pupil dilations, induced by different trial conditions (cue or target types), are compared within the same attention network. In all panels, black dotted lines represent the target onset timing, while red dotted lines represent the response timing. Shaded regions represent the standard error of the mean.

The Benjamini-Hochberg procedure^31^ is applied to control the false discovery rate (FDR) of multiple comparisons (e.g., see Fig. 1.C).

When evaluating the relationship between two variables at the intra- or inter-individual level, linear regressions are conducted using the Theil-Sen estimator;^32^ this provides the advantage of being insensitive to outliers compared to ordinary least squares regression. The significance test on the estimated regressor is performed using a one-sample *t-*test with null hypothesis set to zero.

### Validation

Several validation steps were taken to ensure the quality of the data used in the analysis, with particular attention to two potential sources of bias: 1. Participant exclusion and 2. Trial rejections.

As previously described, the current datasets are part of a larger longitudinal study. Although participants were included based on the availability and quality of pupil data, we were able to compare behavioral variables between the current sample (*n* = 45 controls; *n* = 25 TBI) and the full cohort (*n* = 81 controls; *n* = 70 TBI). Average RTs across all cue and target types and network effects were consistent between the two samples for both control and TBI groups (Welch’s *t*-tests control: lowest *p >* 0.23; TBI: lowest *p >* 0.16).

To assess whether pupil quality-based trial rejections biased behavioral means, we compared behavioral variables (RTs and network effects) calculated from all correct trials with those calculated from the subset of correct and non-rejected trials. Results were consistent across both control and TBI groups. Moreover, within each cohort, all trial types (defined by cues or targets) were equally affected by pupil-based trial rejections.

We also examined potential biases in pupil data quality by comparing the number of blinks and saccades per trial across cohorts. Since these values were comparable between control and TBI participants, we ruled out this concern. However, not surprisingly, the control cohort had significantly fewer rejected trials compared to TBI, 243.2 ± 67.4 against 206.4 ± 66.8 (Welch’s *t-*test: *t* (50.1) = 2.2, *p* < 0.05), despite similar accuracy levels (Welch’s *t-*test: *t*(68.0) = 0.1, *p* > 0.1) defined as the number of correct trials divided by the total number of trials, see Supplementary Table S1. Specifically, the control group had fewer rejected trials for each individual condition (Welch’s *t*-tests: *p* < 0.05). Moreover, within each cohort, the number of trials was comparable across cue types and across target types, implying that trial rejection was not biased toward any specific cue or target type, and ensuring balanced representation for each condition.

## Results

### Demographics and ANT behavior characteristics

For the analysis of pupil data, *n* = 45 adults over the age of 18 (*n* = 16 females, age at test = 40.5 years ±15.2 S.D.) with no history of neurological conditions comprised the control group. Twenty-five adults aged 18 and over (*n* = 9 females, age at test = 48.1 years ±19.1 S.D.), who had sustained a complicated mild, moderate, or severe TBI within 7 months of their testing, on average, comprised the TBI group. The Glasgow Coma Scale (GCS) of the participants in the TBI group ranged from complicated mild (>12, *n* = 16) to moderate/severe (<12, *n* = 9), (see Table 1).

Due to non recorded or poor quality data, *n* = 10 control and *n* = 2 TBI participants were excluded from EEG analysis (see EEG data processing and quality checks). Additional information on mechanism of injury and radiological findings for the participants in the combined pupil and EEG data analysis can be found in Supplementary Table S2.

The control group, when compared to the TBI group, demonstrated significantly faster RTs overall, 594.2 ± 85.1 ms compared with 682.2 ± 92.3 ms (Welch’s *t*-test: *t*(46.4) = −3.9, *p* < 0.001), see Supplementary Table S1. The faster RTs in controls were consistent across all trial conditions, including no-cue, center-cue, spatial-cue, incongruent target, and congruent target (Welch’s *t-*tests: *p* < 0.001 for all cases). In addition, the control group demonstrated stronger effects for the executive network in comparison to the TBI cohort (Welch’s *t*-test: *t*(50.4) = −3.0, *p* < 0.005), suggesting impaired conflict resolution in TBI, however, there was no difference in the magnitude of the effects of the alerting and orienting network (Welch’s *t-*tests: *p* > 0.1), consistent with prior reports.^12,20^

### Pupil dilation during the ANT

In Figure 1, we display pupil dilation in a single control (Panel A), in all control participants (Panel B, *n* = 45), and lastly the group averages of all controls (Panel C). In all panels, we separately display the cue-aligned (left) and response-aligned (right) pupil dilation.

### Relationship to cue onset

Following a center-cue, which informs on target timing, we note a linear and constant expansion. Contrastingly, the spatial-cue, which informs on both target location and timing, elicited a biphasic dilation pattern, with an initial expansion, a temporary plateau near target onset, and a second rise (see Fig. 1.C, Orienting). In the absence of a cue, pupil dilation remains flat until after the target has been displayed (see Fig. 1.C, Alerting). When comparing trials with center and no-cue, differences are visible beginning at cue onset and extending until after the peak. In contrast, comparisons between center and spatial-cue trials revealed a significant difference only at cue onset, with spatial-cues prompting a more rapid dilation (paired *t*-test: *p* < 0.05 after FDR correction).

In response-aligned group averages, the peak dilation amplitude varied significantly across conditions (smallest peak for trials with no-cue: 10.3 ± 1.6 AU; center-cue: 12.9 ± 1.5 AU; spatial-cue: 13.7 ± 1.5 AU) and occurred at different latencies (longest for no-cue: 722 ms post-response; center-cue: 684 ms; spatial-cue: 672 ms), as revealed by a one-way repeated-measures ANOVA (*F*(2, 88) = 9.6, *p* < 0.001).

### Relationship to target onset

Pupil dilation patterns differ significantly between congruent and incongruent targets (paired *t*-test: *p* < 0.05 after FDR correction; see Fig. 1.C, Executive). In the cue-aligned configuration, incongruent targets elicited a larger peak amplitude and longer latency (Peak, paired *t-*test: *t(44)* = −6.1, *p* < 10^−6^; latency, paired *t*-test: *t(44)* = −4.9, *p* < 10^−4^). When aligned to the response, only the amplitude difference remained significant (Peak, paired *t*-test: *t(44)* = −6.0, *p* < 10^−6^; Latency, paired *t-*test: *t(44)* = −0.7, *p* > 0.1).

Across individuals, the average RT is negatively related to the average pupil dilation peak amplitude; this did not meet statistical significance. However, within individuals, trial-by-trial analyses revealed that smaller dilation amplitudes were linked to faster responses in 22% of participants with significant positive slopes (linear regressions: Pearson’s *r* ∈ [0.08, 0.46], *p* < 0.05, flat slopes in the remaining cases). Trial-by-trial, the amplitude of the pupil dilation, following cue or target, is negatively correlated to baseline pupil size (linear regressions: Pearson’s *r* ∈ [−0.64, −0.08], *p* < 0.05 for 96% of the cases).

Across individuals, pupil baseline, peak amplitude, and peak latency were all unrelated to participants’ age.

### Relationship to TBI

**FIG. 2.**
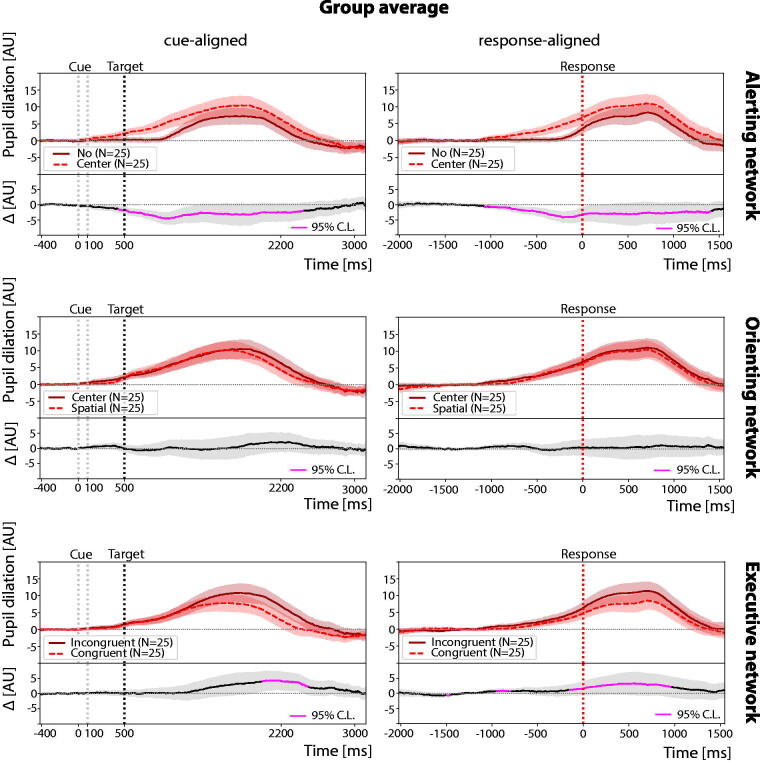
Group average pupil dilation across cue- and response-aligned trials in TBI participants, *n* = 25. Pupil dilations, induced by different trial conditions (cue or target types), are compared within the same attention network. In all panels, black dotted lines represent the target onset timing, while red dotted lines represent the response timing. Shaded regions represent the standard error of the mean. TBI, traumatic brain injury.

In Figure 2, we report the cue- and response-aligned pupil dilation patterns averaged across all participants with TBI (*n* = 25). The pattern closely follows that of controls. At the individual level, 84% of TBI participants (*n* = 21/25), compared with 100% of controls, exhibited a significant increase in pupil dilation during performance of the ANT (one-sample *t*-tests: *p* < 0.05).

For the TBI cohort, we again observed that average pupil dilation is stimulus-dependent (ANOVA: *F*(2, 48) = 5.8, *p* < 0.01). Notably, in the response-aligned group averages, the average peak dilation was smallest for the no-cue condition (8.3 ± 2.2 AU) and occurred latest, 718 ms postresponse. Unlike controls, TBI participants showed the largest peak in the center-cue condition (11.1 ± 2.5 AU) at 702 ms, while the spatial-cue condition produced an intermediate amplitude (10.4 ± 2.5 AU) and the shortest latency, 686 ms.

Compared to controls, two key differences in pupil dilation patterns were observed in individuals with TBI: 1. The absence of a biphasic response following spatial-cues (see Fig. 2, Orienting), and correspondingly, no significant difference between center and spatial-cue trials; 2. less widespread differences between incongruent and congruent trials.

**FIG. 3.**
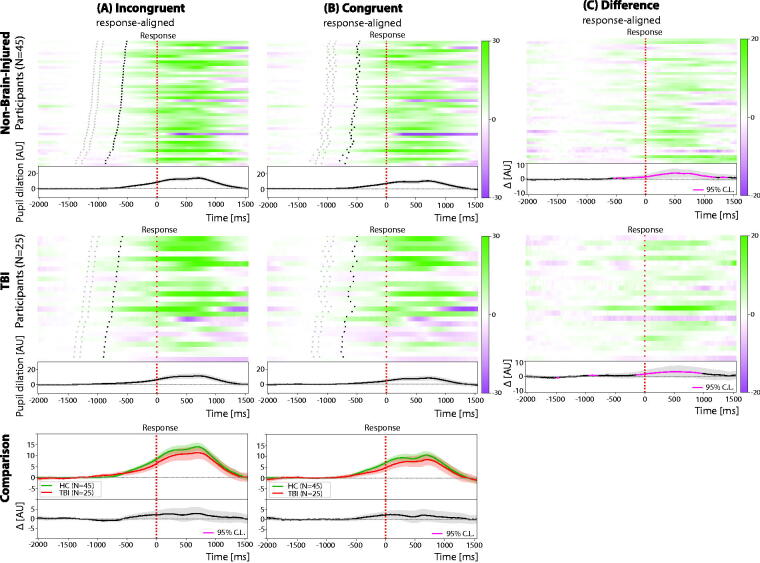
Pupil dilation across response-aligned trials in control (top row) and TBI (middle row) participants. Group average pupil dilation traces are shown separately across target types (bottom row). The difference raster plots (Panel C) are obtained by subtracting the raster plots with congruent trials (Panel B) from the raster plots with incongruent trials (Panel A). Participants in the raster plots are sorted according to increasing mean reaction time in incongruent trials from top to bottom. In all panels, black dotted lines represent the target onset timing, while red dotted lines represent the response timing. Shaded regions represent the standard error of the mean. TBI, traumatic brain injury.

When directly comparing pupil dilation for different target types in response-aligned trials between groups (see Fig. 3), TBI exhibited lower overall amplitudes, although these differences did not reach statistical significance (Welch’s *t*-test: *p* > 0.05 after FDR correction).

Across individuals, the average RT is significantly related to the average pupil dilation peak amplitude (linear regressions: Pearson’s *r* ∈ [−0.65, −0.57], *p* < 0.05), with faster individuals showing greater peak amplitudes; while a similar pattern was noted in controls, it did not meet statistical significance.

Within-individuals, the amplitude of the pupil dilation is related to trial-by-trial performance with smaller dilation associated with faster performance in 15% of participants (linear regressions: Pearson’s *r* ∈ [0.12, 0.34], *p* < 0.05, flat slopes in the remaining cases); we note this in 22% of controls. Similar to controls, trial-by-trial pupil dilation amplitude following cue or target onset was negatively correlated to baseline pupil size, albeit only in 88% of the participants (linear regressions: Pearson’s *r* ∈ [−0.55, −0.04], *p* < 0.05).

### Pupil dilation patterns and relationship to simultaneously recorded ERPs

**FIG. 4.**
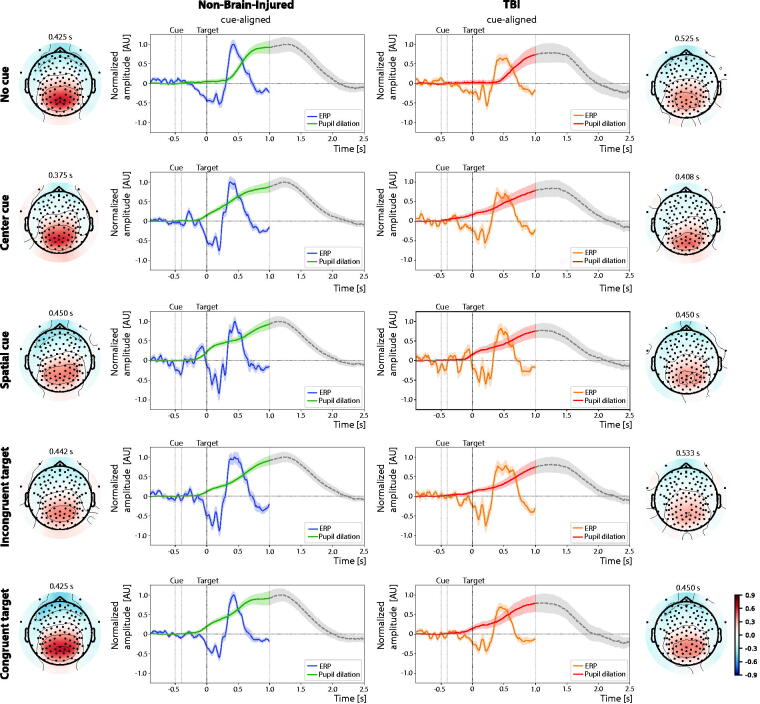
Grand-averaged cue-aligned pupil dilation and P3 event-related potentials (ERP, at electrode Pz) for (left) control and (right) TBI cohorts, shown separately for each cue and target type. Each control P3 ERP and pupil dilation signal is normalized to unit peak amplitude, while the TBI signals are scaled using the same normalization factors to maintain the relative amplitude relationships. EEG topographic maps corresponding to the time of P3 amplitude peak in each condition are also shown. Pupil dilation traces are greyed out beyond 1 s post-target to emphasize the differing time scales of the two physiological signals. In all panels, black dotted lines represent the target onset timing while shaded regions represent the standard error of the mean. ERP, event-related potential; TBI, traumatic brain injury; EEG, electroencephalography.

In Figure 4, we display grand-averaged cue-aligned pupil dilations overlaid to P3 ERPs (at electrode Pz) for control (*n* = 35) and TBI (*n* = 23) cohorts. Each cue and target condition is displayed separately on a different row. Signals for the control cohort have been normalized to unit peak amplitude (i.e., maximum amplitude set to 1 AU), while the TBI cohort signals have been normalized with the corresponding scaling factors to preserve the relative amplitude. Normalized cohort-level EEG topographic maps are extracted at the time of maximum P3 ERP amplitude and are also shown for each condition.

The ERP results replicate findings from a larger cohort study,^12^ specifically demonstrating attenuated P3 amplitudes and delayed P3 peak latency in individuals with TBI, and a reduced P3 amplitude in the congruent condition relative to the incongruent condition across both TBI and control cohorts. The EEG topographic maps reveal similar spatial distributions at the latency of the P3 ERP peaks, which occur at different post-target times (note the delays in TBI participants). As expected, the presence of a visual cue (center or spatial) produces an observable time-locked effect between cue and target onset, a feature absent in the no-cue condition.

Overlaying both pupillary responses and ERP responses, we note that the former are temporally delayed relative to the ERP components. This delay is evident when comparing the timing of the cue-related responses for all conditions. For instance, while the P3 ERP shows a faster cue-locked response, the corresponding change in pupil diameter occurs noticeably later, highlighting this consistent lag in physiological response timing.

When examining the relationship between peak pupil dilation and P3 amplitude within each cohort, a positive linear association was found exclusively in the TBI group. Notably, significant correlations emerged in the spatial-cue and congruent conditions (Pearson’s *r* = 0.36 and 0.50, respectively; *p* < 0.05).

## Discussion

In this study, we examined pupil dilation during the ANT in individuals with TBI and controls. TBI participants exhibited preserved accuracy but slower RTs, in addition to attenuated pupil responses. We further assessed the relationship between simultaneously recorded pupillary and visual evoked responses in a subset of participants with both measures. While both pupil dilation and visual P3 event-related potential amplitude were reduced in TBI, these measures showed a positive relationship that was not observed in controls.

### Pupil dilation as an indicator of cognitive load and effort

Our findings reinforce the established relationship between pupil dilation and cognitive load.^5,8^ In controls, pupil dilation increased proportionally to attentional demands; pupil dilation peaks later for incongruent versus congruent targets, reflecting the increased effort required for conflict resolution.^33^ In addition, cues elicited stronger and more sustained pupil dilation, suggesting heightened engagement of alerting and orienting systems. These findings align with prior research demonstrating that spatial-cues enhance cognitive preparation and facilitate more efficient response strategies.^17^

At the individual level, baseline pupil size negatively correlated with peak dilation, consistent with autonomic constraints on dilation capacity.^34^ A larger baseline pupil size may reflect higher baseline (tonic) arousal, limiting task-evoked (phasic) dilation in response to task demands.^35^ Only 22% of controls exhibited a trial-by-trial correlation between pupil dilation and RT, suggesting that pupil-linked cognitive control varies across individuals and is influenced by factors such as arousal regulation and autonomic function.^33^

### Altered pupillary response in TBI

Consistent with previous findings related to the ANT,^12,20^ TBI participants maintain high accuracy but exhibit prolonged RTs; this could indicate increased mental effort to sustain performance, particularly in incongruent trials. However, despite the greater cognitive effort, pupil dilation is reduced compared to controls, suggesting autonomic dysfunction and impaired LC-NE signaling.^33,35,36^ Blunted response may reflect a diminished ability to modulate arousal in response to cognitive demands, contributing to greater cognitive fatigue and attentional instability.^9^

A notable difference between TBI and controls is the lack of sustained pupil dilation. In controls, incongruent trials evoke larger, sustained pupil dilation, reflecting continuous cognitive engagement and sustained attentional control.^5^ In contrast, TBI participants exhibit a weaker and less sustained dilation response, consistent with reduced top-down attentional regulation and effort maintenance over time.^3,37^

### Increased coupling between P3 ERP and pupil dilation in TBI: a potential compensatory mechanism

Research in control individuals has reported varied findings regarding the trial-by-trial relationship between P3 ERP amplitude and pupil dilation, with some studies identifying strong correlations in the context of emotionally significant stimuli^16^ or under high cognitive load;^38^ others found no direct relationship.^13–15^ The strength of this relationship appears to depend on task complexity, engagement, cognitive effort, and individual differences in attention regulation.^13,38,39^

In our study, both P3 amplitude and pupil dilation are reduced in TBI, yet they are more tightly correlated than in controls. This may suggest that TBI patients rely more heavily on shared compensatory mechanisms, wherein autonomic engagement plays a greater role in sustaining cognitive function.^40–42^ Studies examining other autonomic markers (e.g., heart rate variability) in TBI similarly show stronger EEG-autonomic coupling, implying that compensatory processes extend beyond cortical mechanisms to include subcortical arousal regulation.^2,43^

A possible unifying mechanism suggested by our findings is the underactivation of frontal cortical monitoring resources in the TBI subjects. In non-human primates, pupillary dilation in response to cognitive load is encoded by frontal cortical neurons in Brodmann area 8 and frontal eye field.^44^ In TBI subjects, a chronically down-regulated frontal cortical tone^45^ likely underlies a lack of monitoring feedback to subcortical arousal regulation systems. Notably, frontal eye fields have reciprocal connections with neurons within the central lateral nucleus,^46^ the key thalamic component of the anterior forebrain mesocircuit model of impaired frontal function after TBI.^47^ These neurons are, in principle, thalamic targets of the locus coeruleus^48^ and have known direct modulation of visual evoked responses.^49^ In addition, anterior cingulate neurons have reciprocal projections to locus coeruleus and coactivate in novel environments.^50^ Chronic impairment of frontal cortical monitoring of arousal regulation could explain the tighter correlations we observed: in TBI subjects, stimulus-driven activation from the brainstem, thalamic, and posterior cortical orienting and alerting systems more strongly drives frontal activation. In contrast, in controls, the frontal systems are likely more tonically active, reducing the prominence of this effect.

To the best of our knowledge, no previous studies have explicitly tested the relationship between P3 ERP amplitude and pupil dilation in TBI individuals. In controls P3 and pupil dilation can be modulated independently, allowing for adaptive cognitive engagement without excessive autonomic activation.^51^ However, in TBI, the reduced ability to flexibly allocate neural resources may force a stronger reliance on autonomic regulation to sustain performance, particularly in tasks requiring sustained attention and response inhibition. Understanding these mechanisms could provide insights into neurophysiological markers of cognitive resilience and impairment in TBI.

### Limitations of the current study

This study has several limitations that should be considered when interpreting the findings.

First, the lack of a chin-resting system may have introduced measurement noise due to small head movements, leading to data loss and potential variability in pupil dilation estimates. Future studies should employ high-resolution, head-stabilized pupillometry to enhance precision. In addition, while our sample included individuals with complicated mild to severe TBI, the heterogeneity of TBI presents challenges in generalizability, and subgroup analyses may have been underpowered. Larger, well-stratified cohorts will be necessary to better understand how injury severity, lesion location, and chronicity influence EEG-pupil interactions. Another key limitation is the cross-sectional design, which does not allow us to determine how EEG-pupil coupling evolves over time or whether it predicts cognitive recovery. Longitudinal studies will be essential for tracking changes in autonomic-cognitive regulation throughout the recovery process.

Moreover, the study was conducted in a controlled laboratory setting using the ANT, which ensures internal validity but may not fully capture real-world attentional challenges in TBI patients. Future research should examine EEG-pupillometry interactions in naturalistic cognitive tasks to improve clinical applicability. In addition, the task-dependent nature of P3-pupil relationships remains a consideration, as these findings may not generalize to memory, decision-making, or social cognition tasks. Future studies might consider isolating components of the ERP, as was previously done,^38^ before relating it to pupil dilation.

Furthermore, factors such as medication use, fatigue, and baseline autonomic state may have influenced pupil and EEG responses, highlighting the need for future studies to incorporate additional physiological control measures (e.g., heart rate variability, galvanic skin response) to disentangle cognitive versus autonomic contributions. Lastly, while we demonstrate stronger EEG-pupil coupling in TBI, the underlying neural mechanisms remain unclear. Whether this reflects compensatory recruitment of autonomic pathways, impaired cortical control over arousal, or broader neural inflexibility requires further investigation. Future research integrating fMRI, diffusion tensor imaging, and neuromodulation (e.g., TMS, vagus nerve stimulation) could help clarify the neural circuitry underlying EEG-pupil interactions in TBI and inform more targeted rehabilitation approaches.

### Implications for future research in brain injury

Our findings suggest that integrating EEG and pupil dilation measures could provide a sensitive, multimodal biomarker of cognitive load, effort regulation, and compensatory processing in TBI. While standard neuropsychological tests may fail to detect subtle impairments, EEG-pupillometry interactions could reveal how individuals allocate cognitive and autonomic resources in real-time, offering potential applications in early diagnosis, recovery monitoring, and personalized rehabilitation planning.^41,52–54^

Future research should explore:

1. How EEG-pupillometry interactions evolve over time in TBI: e.g., can these measures predict cognitive recovery trajectories?
2. Whether this coupling varies by injury severity: e.g., do mild versus severe TBI patients show different patterns?
3. How rehabilitation interventions influence EEG-pupil dynamics: e.g., can cognitive training or neuromodulation alter this compensatory mechanism?

In addition, differentiating TBI subtypes (e.g., focal vs. diffuse) using EEG-pupil biomarkers could enhance diagnostic precision and treatment personalization.^55^ Given the evidence that EEG-pupil correlations are stronger in TBI, further research should investigate how this relationship changes across recovery stages and whether fluctuations in these markers signal cognitive instability or emerging compensatory mechanisms.^37,52^

## Transparency, Rigor, and Reproducibility Statement

All analyses were conducted using established, peer-reviewed methods, and results are reported in accordance with current field standards for statistical transparency and reproducibility. Data preprocessing steps, statistical models, and code used for analysis have been documented in detail to ensure reproducibility. Deidentified data and custom code are available upon reasonable request to the corresponding author, in compliance with institutional guidelines and participant consent.

## Abbreviations Used

ANT: Attention Network Test
ERPs: Event-related potentials
EEG: Electroencephalography
LC–NE: locus coeruleus–norepinephrine
RTs: Reaction Times
TBI: Traumatic brain injury
