# Supplementary material for: Coupling of Event-Related Potential and Pupil Dilation as a Compensatory Marker of Executive Attention in Traumatic Brain Injury
Source: Neurotrauma Rep. 2025 Aug 26;6(1):706–19. doi: 10.1177/2689288X251370997 (PMC12416529; doi:10.1177/2689288X251370997)
Supplement: Supplementary Data [file 2689288x251370997_supp_data.docx]

**SUPPLEMENTARY MATERIAL**

**SUPPLEMENATARY FIGURE LEGEND**

**
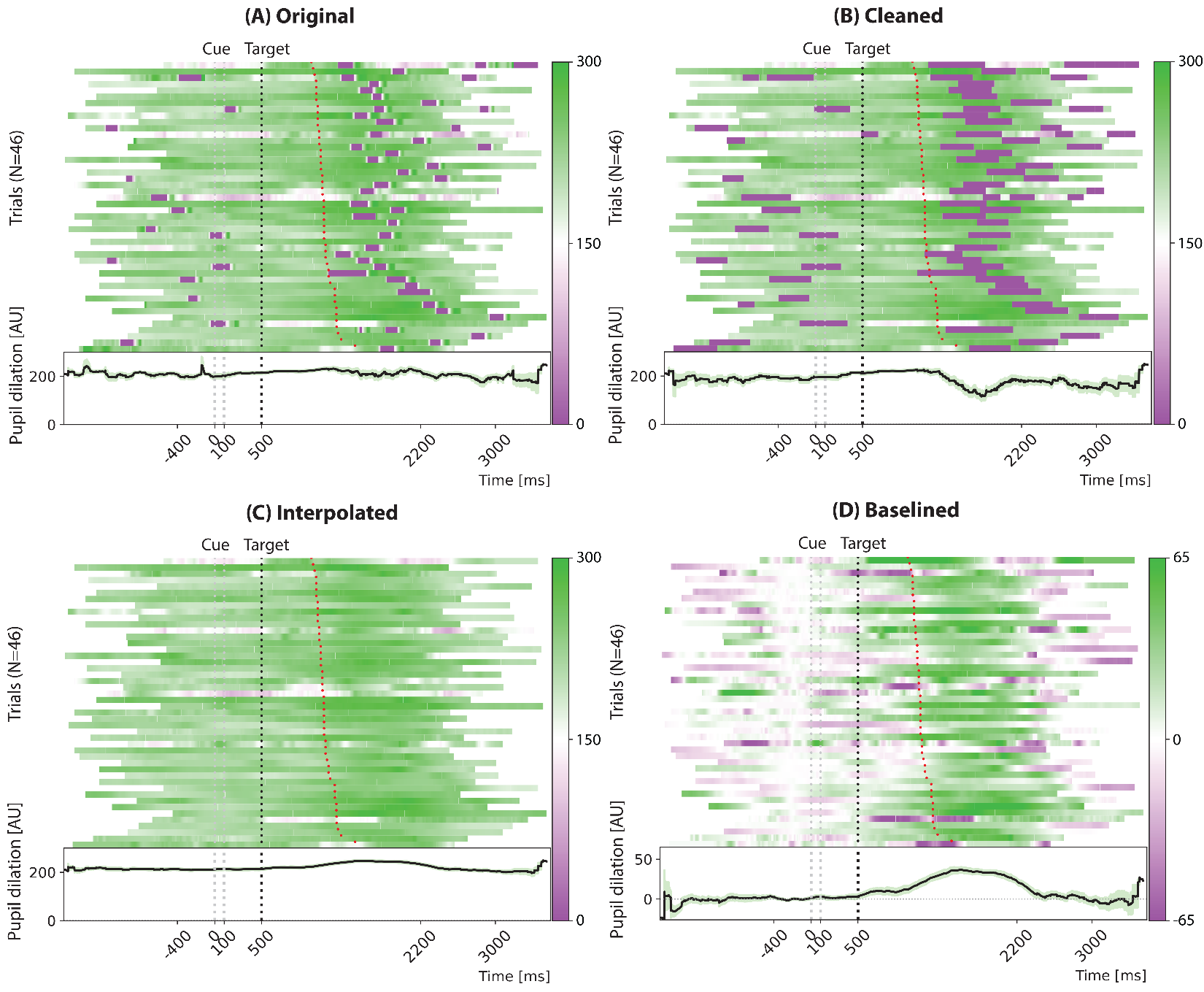
**

**Supplementary Figure S1:** Raster plots showing cue-aligned congruent trials with center-cue for one of the participants included in the pupil analysis. Starting from the left: (A) all trials are shown with their raw signals, with purple segments representing the measured blinks; (B) blink segments are extended as described in Pupil data processing and quality checks to remove edge effects; (C) the missing values are filled in via linear interpolation; (D) each trial is independently baselined. In all panels, black dotted lines represent the target onset timing, while red dotted lines represent the response timing. Note the change in color meaning for the baselined trials in Panel D.

**SUPPLEMENTARY TABLE**

|  | **Non-Brain Injured** | | **TBI** | | **p** |
| --- | --- | --- | --- | --- | --- |
|  | **Mean** | **S.D.** | **Mean** | **S.D.** |  |
| **Reaction Times [ms]** | | | | | |
| **No Cue** | **628.3** | **84.3** | **710.6** | **89.2** | **< 10⁻³** |
| **Center Cue** | **600.8** | **81.9** | **692.1** | **93.5** | **< 10⁻³** |
| **Spatial Cue** | **553.7** | **93.6** | **644.8** | **97.3** | **< 10⁻³** |
| **Incongruent Target** | **641.1** | **94.6** | **743.6** | **97.9** | **< 10⁻³** |
| **Congruent Target** | **549.0** | **78.0** | **625.6** | **89.0** | **< 10⁻³** |
| **All Trials** | **594.2** | **85.1** | **682.2** | **92.3** | **< 10⁻³** |
| **Network Effects [ms]** | | | | | |
| **Alerting** | **27.5** | **24.5** | **18.6** | **26.1** | **> 0.1** |
| **Orienting** | **47.1** | **28.0** | **47.2** | **20.2** | **> 0.1** |
| **Executive** | **92.1** | **35.5** | **118.1** | **34.9** | **< 0.005** |
| **Accuracy [%]** | | | | | |
| **All Trials** | **97.4** | **4.8** | **97.3** | **2.6** | **> 0.1** |
| **Number of Trials (Pupil data)** | | | | | |
| **No Cue** | **81.0** | **22.2** | **68.2** | **22.7** | **< 0.05** |
| **Center Cue** | **81.0** | **22.5** | **68.4** | **22.8** | **< 0.05** |
| **Spatial Cue** | **81.3** | **22.9** | **69.9** | **21.7** | **< 0.05** |
| **Incongruent Target** | **120.0** | **33.4** | **100.2** | **34.8** | **< 0.05** |
| **Congruent Target** | **123.2** | **33.8** | **106.2** | **32.7** | **< 0.05** |
| **All Trials** | **243.2** | **67.4** | **206.4** | **66.8** | **< 0.05** |

**Supplementary Table S1**: Summary of reaction times, network effects, accuracy, and number of trials for non-brain-injured and TBI cohorts. The p-values for the Welch’s t-tests are reported in the last column. The control group showed faster reaction times and better conflict resolution (executive network). Overall, the TBI cohort had similar accuracy but consistently fewer trials per type compared to the control group.

| **ID** | **Sex** | **Age** | **Mechanism of**  **Injury** | **Radiologic Findings:**  **Type** | **Radiologic Findings: Location** | **Glasgow**  **Coma Scale**  **(GCS)** |
| --- | --- | --- | --- | --- | --- | --- |
| 030* | M | 72.7 | Fall head trauma  (from standing) | SAH; SDH; bilateral nondisplaced parietal fractures | R frontal lobe | Complicated mild |
| 055* | M | 22.2 | Motor vehicle hit biker | SAH | L parietal lobe | Complicated mild |
| 003 | M | 57.7 | Motor vehicle hit motorcyclist | SDH; IPH | R frontal lobe | Complicated mild |
| 017 | F | 48.7 | Motor vehicle hit pedestrian | SAH | Bilateral frontal lobes | Complicated mild |
| 024 | M | 50.8 | Fall head trauma  (from stairs) | EDH (mild mass effect); nondisplaced parietal-temporal fracture; SAH layering; low-density extra-axial fluid | R frontal, temporal, and parietal sulcal regions | Complicated mild |
| 025 | F | 74.4 | Non-motor vehicle hit pedestrian | SDH; SAH | L frontal lobe; L cerebral hemisphere sulci | Complicated mild |
| 027 | M | 68.6 | Blunt head trauma (punch) | SAH; occipital subcutaneous hematoma | R parasagittal frontal and occipital regions | Complicated mild |
| 034 | M | 82.8 | Fall head trauma (unspecified) | SDH; ventricular compression/enlargement | R cerebral convexity | Complicated mild |
| 038 | F | 52.8 | Fall head trauma  (from standing) | SDH | R frontoparietal convexity | Complicated mild |
| 039 | M | 56.7 | Motor vehicle hit biker | SDH | Posterior falx cerebri | Complicated mild |
| 041 | F | 52.0 | Motor vehicle hit pedestrian | SDH; IPH | R temporal region | Complicated mild |
| 044 | M | 63.3 | Non-motor vehicle hit pedestrian | SDH | R anterior falx cerebri | Complicated mild |
| 045 | F | 50.6 | Fall head trauma  (ice skating) | SDH | R falx cerebri and tentorial region | Complicated mild |
| 047 | F | 31.5 | Non-motor vehicle hit pedestrian | IPH | R cerebral hemisphere | Complicated mild |
| 058 | M | 79.2 | Non-motor vehicle hit pedestrian | SDH; hemorrhagic contusion | R frontal lobe; R cerebral convexity | Complicated mild |
| 065 | F | 58.4 | Fall head trauma  (from standing) | SAH; SDH; IPH; multicompartmental hemorrhage; minimal shift; temporal bone fracture | L frontal, parietal, and occipital lobes | Complicated mild |
| 013 | M | 38.2 | Motor vehicle hit motorcyclist | SAH; DAI; punctate hemorrhages; contusions | Sup. L frontal region; R temporal lobe | Moderate |
| 014 | M | 27.1 | Motor vehicle hit motorcyclist | SAH | R frontoparietal region | Moderate |
| 023 | M | 19.0 | Blunt head trauma (punch and kick) | IPH | R posterior temporal region | Moderate |
| 005 | F | 33.3 | Blunt head trauma (punch) + Fall head trauma (from standing) | SAH; SDH; petechial hemorrhages | R frontal and temporal lobes | Severe |
| 008 | M | 41.5 | Blunt head trauma (baseball bat) | EDH; extra-axial bleed; hemorrhagic contusions; fractures | L temporal lobe | Severe |
| 009 | F | 23.6 | Motor vehicle driver hit wall | SDH | L cerebral convexity | Severe |
| 046 | M | 38.1 | Fall head trauma  (from bike) | SAH | R frontoparietal region | Severe |
| 068 | M | 20.7 | Motor vehicle hit pedestrian | Uknown | Uknown | Severe |

**Supplementary Table S2**: Additional information regarding participants with TBI included in the analysis. Participant IDs with (*) indicate exclusion from the combined Pupil and EEG analysis. The radiologic findings are extracted from CT scans. Acronym explanation: (SAH) subarachnoid hemorrhage; (SDH) subdural hematoma; (IPH) intraparenchymal hemorrhage; (EDH) epidural hematoma; (DAI) diffuse axonal injury.
